# The Waste of the Household

**Published:** 1891-12

**Authors:** 


### THE WASTE OF THE HOUSEHOLD.

Mr. Edward Atkinson, if not exactly making two blades of grass grow where one grew before, like Dr. Johnson's typical benefactor, is trying hard to make $1 in food preparation do the work of $2, which would be a great deal better. He declares in a recent publication the cost of food material absorbs half of the income of nine-tenths of our people ; the waste of food exceeds cost of all the cloth made in our factories ; and that we consume a pound of coal for every pound of food that is cooked for our breakfast and dinner tables. We cook in the United States, he says, at least $3,000,000,000 worth of meat, fish, grain and vegetables in a year, and of that sum we lose $1,000,000,000 by bad cookery.

Being a light eater, and subsisting on $1 a week for food alone, Mr. Atkinson prefers the oil or gas stove to the ponderous "range" or "cook-stove," which he thinks an invention of Satan. He adds this calculation : I gave a seven-course dinner to my whist club friends, including oranges and coffee, which cost 13 cents each for the food material. I gave a dinner of four courses, soup, fish, meat and vegetables, and mush with molasses for desert, to nine of the poorer students at Harvard who want to economize ; there were also three others ; each had a pound and a half of strong food and the cost of twelve was 61 cents. And yet the authorities made us pay $1 each for our commencement dinner of four courses a month ago !

The miracle of the five loaves and two small fishes is not repeated yet by Mr. Atkinson, but he is making approaches towards it. He says, and no doubt very truly, that a laborer whose wife knows how to choose meat as well as to cook it, can live on $1 a week, and much better than the average man fares, though this would compel her to buy the poorer kinds of beef. But for $2 a week the laboring man can buy the best, always excepting canvass-back ducks and tenderloin beef ; and that, in Boston, a hard-working man can live well for 20 cents a day, and a woman at 13¾ cents. I have no doubt of this, for I know a family which lives better than most persons, so far as food goes, for $1.11 a week, cooking included. But this requires great care and intelligence in buying and preparing the food.—*Boston Advertiser.*

